# Supplementary material for: A Comprehensive Assessment of Ultraviolet-Radiation-Induced Mutations in Flammulina filiformis Using Whole-Genome Resequencing
Source: J Fungi (Basel). 2024 Mar 20;10(3):228. doi: 10.3390/jof10030228 (PMC10971301; doi:10.3390/jof10030228)
Supplement: Supplementary file 1 [file jof-10-00228-s001.zip › Supplementary Material S8/KEGG annotation/out/64381550635650.os/KO/out_map/map04350.html]

KEGG PATHWAY: TGF-beta signaling pathway - Reference pathway


|  |  |
| --- | --- |
| **TGF-beta signaling pathway - Reference pathway** |  |

[
Pathway menu
| Organism menu
| Pathway entry
| Show description
| User data mapping
]

The transforming growth factor-beta (TGF-beta) family members, which include TGF-betas, activins and bone morphogenetic proteins (BMPs), are structurally related secreted cytokines found in species ranging from worms and insects to mammals. A wide spectrum of cellular functions such as proliferation, apoptosis, differentiation and migration are regulated by TGF-beta family members. TGF-beta family member binds to the Type II receptor and recruits Type I, whereby Type II receptor phosphorylates and activates Type I. The Type I receptor, in turn, phosphorylates receptor-activated Smads ( R-Smads: Smad1, Smad2, Smad3, Smad5, and Smad8). Once phosphorylated, R-Smads associate with the co-mediator Smad, Smad4, and the heteromeric complex then translocates into the nucleus. In the nucleus, Smad complexes activate specific genes through cooperative interactions with other DNA-binding and coactivator (or co-repressor) proteins.

|  |  |
| --- | --- |
| Reference pathway | 100% |
